# Deep Active Learning *via* Open-Set Recognition

**DOI:** 10.3389/frai.2022.737363

**Published:** 2022-02-07

**Authors:** Jaya Krishna Mandivarapu, Blake Camp, Rolando Estrada

**Affiliations:** Department of Computer Science, Georgia State University, Atlanta, GA, United States

**Keywords:** deep learning, active learning, autoencoders, manifold learning, open set recognition

## Abstract

In many applications, data is easy to acquire but expensive and time-consuming to label, prominent examples include medical imaging and NLP. This disparity has only grown in recent years as our ability to collect data improves. Under these constraints, it makes sense to select only the most informative instances from the unlabeled pool and request an oracle (e.g., a human expert) to provide labels for those samples. The goal of active learning is to infer the informativeness of unlabeled samples so as to minimize the number of requests to the oracle. Here, we formulate active learning as an open-set recognition problem. In this paradigm, only some of the inputs belong to known classes; the classifier must identify the rest as *unknown*. More specifically, we leverage variational neural networks (VNNs), which produce high-confidence (i.e., low-entropy) predictions only for inputs that closely resemble the training data. We use the inverse of this confidence measure to select the samples that the oracle should label. Intuitively, unlabeled samples that the VNN is uncertain about contain features that the network has not been exposed to; thus they are more informative for future training. We carried out an extensive evaluation of our novel, probabilistic formulation of active learning, achieving state-of-the-art results on MNIST, CIFAR-10, CIFAR-100, and FashionMNIST. Additionally, unlike current active learning methods, our algorithm can learn even in the presence of out-of-distribution outliers. As our experiments show, when the unlabeled pool consists of a mixture of samples from multiple datasets, our approach can automatically distinguish between samples from seen vs. unseen datasets. Overall, our results show that high-quality uncertainty measures are key for pool-based active learning.

## 1. Introduction

Supervised deep learning has achieved remarkable results across a variety of domains by leveraging large, labeled datasets (LeCun et al., 2015). However, our ability to collect data far outstrips our ability to label it, and this difference only continues to grow. This problem is especially stark in domains where acquiring the ground truth requires a highly trained specialist, e.g., medical imaging. Even in cases where labeled data is sufficient, there may be reasons to limit the amount of data used to train a model, e.g., time, financial constraints, or to minimize the model's carbon footprint.

Fortunately, the relationship between a model's performance and the amount of training data is not linear. There often exists a small subset of highly *informative* samples that can provide most of the information needed to learn to solve a task. In this case, we can achieve nearly the same performance by labeling (and training on) only those informative samples, rather than the entire dataset. The challenge, of course, is that the true usefulness of a sample can only be established *a posteriori*, after we have used it to train our model.

The growing field of *active learning* (AL) is concerned with automatically predicting which samples from an unlabeled dataset are most worth labeling^1^. In the standard AL framework, a selector identifies an initial set of promising samples; these are then labeled by an oracle (e.g., a human expert) and used to train a task network (Gal et al., 2017). The selector then progressively requests labels for additional batches of samples, up to either a percentage threshold (e.g., 40% of the total data) or until a performance target is met. In short, an active learning system seeks to construct the smallest possible training set which will produce the highest possible performance on the underlying task/s.

In this paper, we formulate active learning as an *open-set recognition (OSR) problem*, a generalization of the standard classification paradigm. In OSR, only some of the test inputs are from the trained-upon distribution; the classifier must label the remaining inputs as *out-of-distribution (OOD)*, meaning that they do not match the types of inputs it was trained on. For example, if a network was trained on digit recognition, e.g., using MNIST, then images of animals or vehicles, such as those of CIFAR-10, would be OOD. Here, we view the labeled pool as the training distribution. The unlabeled samples which are similar to the labeled pool are deemed as in-distribution, while the unlabeled samples that are very different from the labeled pool are marked as OOD. Our hypothesis is that the samples most worth labeling are those that are most different from the currently labeled pool (i.e., those deemed OOD) because they contain features which the network has not yet been exposed to. Thus, training on these samples will allow the network to learn these features that are underrepresented in the existing training data. In short, our AL selection mechanism consists of picking unlabeled samples that are OOD relative to the labeled pool.

**Figure 1:**
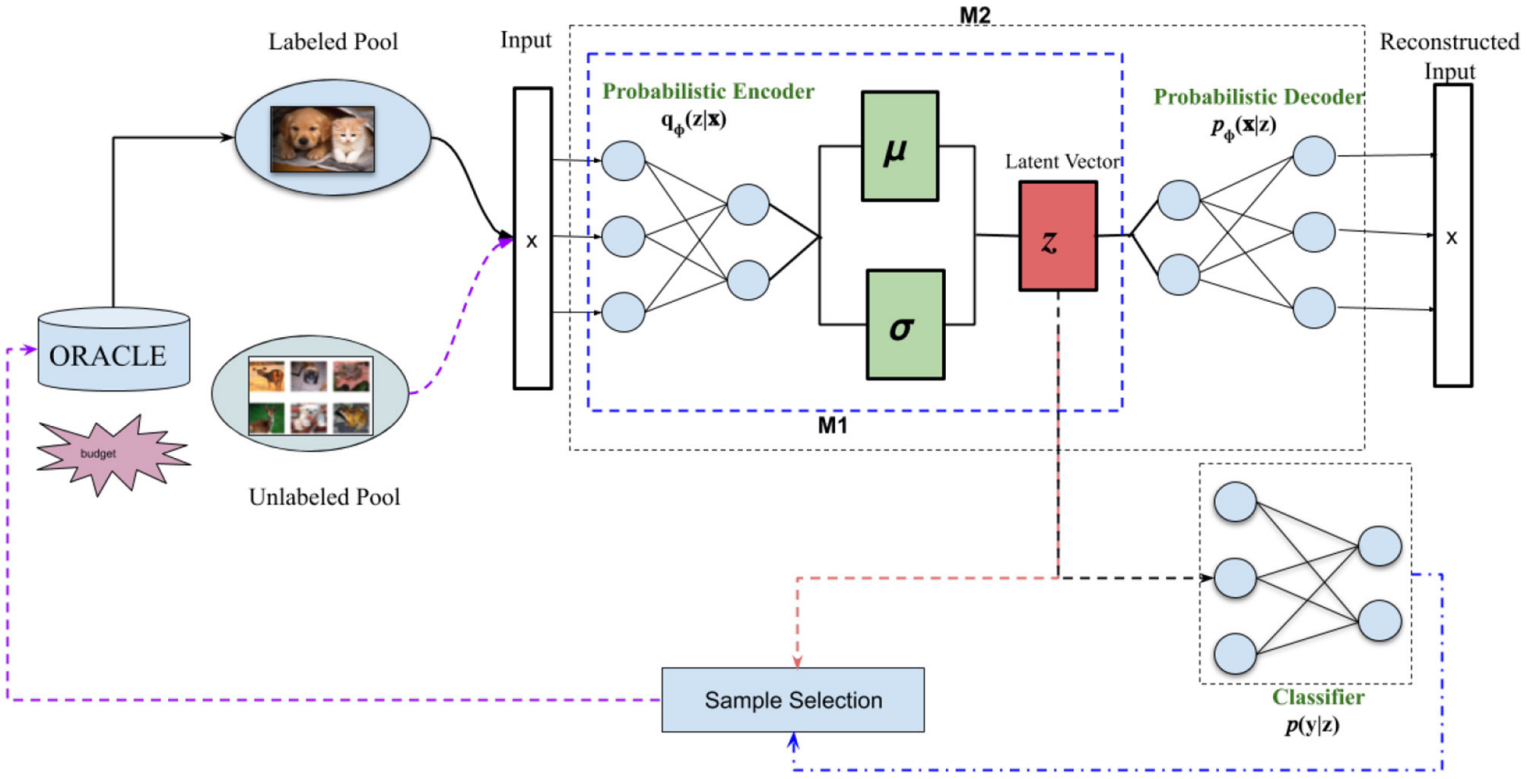
Framework overview: Our proposed active learning system uses open-set recognition to identify which samples from the unlabeled pool to label. Our classifier is a variational neural network (VNN) (Mundt et al., 2019b), which simultaneously reconstructs an input using a probabilistic autoencoder (AE) and classifies it by feeding the AE's latent vector *z* to a linear classifier. We use the VNN's loss function to determine which samples to select from the unlabeled pool (Sample Selection). As in Mundt et al. (2019b), we tested two VNN variants: M1 is trained using only the loss on the latent vector *q*_Φ_(*z*|*x*) and the classifier *p*(*y*|*z*), while M2 also includes the loss on the reconstructed input *p*_Φ_(*x*|*z*). Figure based on similar diagrams in Mundt et al. (2019a) and Sinha et al. (2019).

Figure 1 illustrates our proposed approach. In more detail, our classifier is a variational neural network (VNN) (Mundt et al., 2019b), which produces high-confidence (i.e., low-entropy) outputs only for inputs that are highly similar to the training set. VNNs are explicitly trained to maximize the entropy of their outputs for inputs that differ from the training set; thus, entropy-based confidence measures are more reliable for VNNs than for regular neural networks. Specifically, we use the inverse of this entropy-based confidence measure to select which unlabeled samples to query next. In other words, our selector requests labels for the samples that the classifier is *least confident* about because this implies that the existing training set does not contain items with similar features to them. As we detail in section 4, our OSR-based approach achieved state-of-the-art results in a number of datasets and AL variations, far surpassing existing methods.

The rest of this paper is organized as follows. In section 2, we provide a brief overview of current active learning and open-set recognition methods. In section 3, we present our proposed approach, then detail our experiments in section 4. Finally, we discuss avenues for future work in section 5.

## 2. Prior Work

### 2.1. Pool-Based Active Learning

It has been shown that training samples do not contain equal amounts of useful information (Settles, 2010). Thus, the goal of sampling-based active learning is to learn an ***acquisition function*** that chooses the best data points for which a label should be requested from a large, unlabeled pool of data (Gal et al., 2017). There have been numerous efforts to learn an optimal sampling strategy, and they can be broadly grouped into three major categories (Sinha et al., 2019).

#### 2.1.1. Uncertainty-Based Techniques

Uncertainty-based techniques aim to select samples from the unlabeled distribution about which the current classifier is highly uncertain. Different metrics have been proposed for quantifying how uncertain a model about a sample. Some methods such as Settles (2012), Settles and Craven (2008), Luo et al. (2013), and Joshi et al. (2009) used the entropy of the posterior probability of the model, whereas methods such as Joshi et al. (2009) and Roth and Small (2006) use difference margin between the first and second predicted class to select the samples. Some approaches (Lewis and Catlett, 1994; Lewis and Gale, 1994; Wang et al., 2016) directly use the probability outputs to select the samples. Other methods map the network's outputs to a probability distribution to achieve better sample selection from the unlabeled pool. For example, Yoo and Kweon (2019) proposed a loss-learning module along with regular classifier which can predict the loss for given unlabeled pool image; images with high prediction loss are selected to be labeled by the oracle. Similarly, Gal and Ghahramani (2016) used a Monte Carlo dropout methods to obtain an uncertainty estimate for each sample.

#### 2.1.2. Diversity and Hybrid-Based Methods

Representations-based models aim to maximize the *diversity* in training batches (Sener and Savarese, 2017). For example, Kirsch et al. (2019) used a Bayesian formulation to determine sample diversity, while used gradient embeddings for assessing the similarity between samples. The approach in Shui et al. (2019), on the other hand, formulate sample selection as distribution matching. Hybrid approaches attempt to combine quantifiable uncertainty and diversity in order to select training samples (Li and Guo, 2013). VAAL (Sinha et al., 2019) proposed an adversarial learning based method in which a discriminator is trained along with the task network to discriminate whether an example belongs to the labeled or unlabeled set. In Sener and Savarese (2017), the authors considered active learning as a set-cover problem, one in which a task network is trained using a core-set loss, which is the difference between a task-network's classification error over the labeled set vs. the core-set. DBAL (Gal et al., 2017) approached the active learning problem using Bayesian convolutional neural networks, wherein confidence is measured using variation ratios. In MC-Dropout (Gal and Ghahramani, 2016), the authors proposed to model the uncertainty present in deep networks by interpreting dropout as a type of Bayesian inference in deep Gaussian processes.

### 2.2. Open-Set Recognition

Open-Set Recognition (OSR) refers to the ability of a system to distinguish between types of data it has already seen (the training distribution) from types to which it has not yet been exposed (out-of-distribution (OOD) data). Standard deep neural networks are not suitable for OSR because they often yield high confidence values for inputs which are significantly different from the training classes. This vulnerability has been exploited for adversarial attacks on deep networks, specifically to change a classifier's labels based on imperceptible changes to the input image (Goodfellow et al., 2014). VNNs, on the other hand, are explicitly trained to maximize the entropy of their outputs for samples that differ from those it was trained on, so they achieve OSR results.

More generally, as noted by Geng et al. (2020), existing OSR methods can be subdivided into discriminative-based and generative-based approaches. Discriminative methods modify traditional ML and deep neural networks to tackle the OSR problem. For example, Scheirer et al. (2012) used traditional SVMs with an additional open space risk term, while (Zhang and Patel, 2016) extended sparse classifiers to OSR by modeling the error distribution using Extreme Value Theory (EVT) (Vignotto and Engelke, 2018). Some other discriminative methods use nearest neighbors (Júnior et al., 2017), probability models (Jain et al., 2014; Scheirer et al., 2014; Scherreik and Rigling, 2016), or outlier detection (Bendale and Boult, 2015).

Generative methods primarily use generative adversarial networks (GANs) (Goodfellow, 2016) for OSR. For example, Neal et al. (2018) proposed G-OpenMax by adopting an encoder-decoder GAN architecture for generating samples which are highly similar to training samples yet do not belong to any of the training classes. Following a similar approach, Yang et al. (2019) investigated the open-set human activity recognition problem based on micro-Doppler signatures by using a GAN to generate samples which were highly similar to the target class and forming a negative set out of it. Not all generative approaches use GANs, though. For example, Geng and Chen (2018) proposed a collective, decision-based OSR model by slightly modifying the hierarchical Dirichlet process.

## 3. Methodology

As noted above, our active learning approach selects batches of samples from an unlabeled pool based on the confidence level of its OSR classifier. Below, we first formalize the active learning paradigm we are tackling, then detail our proposed system. In particular, we provide an overview of VNNs and explain how we use their outputs to select new samples to label.

### 3.1. Formal Problem Definition

Formally, a pool-based active learning problem is denoted as *P* = (*C, D*_*train*_, *D*_*test*_), where *C* indicates the number of classes, *D*_*train*_ is the training set, and *D*_*test*_ is the test set, s.t. *D*_*train*_ ∩ *D*_*test*_ = ∅. Let $D_{train}={\left\{\left(x_{i},y_{i}\right)\right\}}_{i=1}^{N}$ consist of *N* i.i.d. data points where only *m* of them are labeled (*m* << *N*). Each sample $x_{i}\in {\mathbb{R}}^{d}$ is a *d*-dimensional feature vector, and *y*_*i*_ ∈ {1, 2, …, *C*} represents the target label. At the start, $D_{train}$ consists of two disjoint subsets: a labeled pool L containing the *m* labeled data points, and an unlabeled pool U which includes the remaining *N* − *m* data points with unknown target labels. We will update both L and U after each query to the oracle. We denote the state of a subset at a given timestep as $L^{t}$ and $U^{t}$, respectively, for *t* ∈ {0, 1, …}. At any given iteration, the budget *b* is defined as total number of samples from the unlabeled pool ($U^{t}$) for which we can request labels from the oracle. As with most active learning papers, in our experiments we simulate the oracle by withholding some of the labels from a standard dataset.

In active learning, we first train a classifier *f*, with parameters θ, on $L^{0}$. Afterwards we select *b* data points from $U^{0}$ using our OSR criterion (see section 3.2). These *b* data points are then sent to the oracle for annotation. The annotated samples are removed from the unlabeled pool and added to the labeled pool, along with their newly acquired target labels. The updated labeled and unlabeled data pools become $L^{1}$, of size *m* + *b*, and $U^{1}$, respectively. Thus, the labeled pool grows in size as training progresses. We continue this process until the size of the labeled pool reaches a predefined limit (40% of *D*_*train*_ in our experiments). Note that, while the above formulation is similar to continual learning, in active learning we assume that samples from all classes are present in both the labeled and unlabeled pools during all iterations. We do not learn new classes in an incremental fashion.

Importantly, unlike other formulations of AL, here we allow for the unlabeled pool U to contain training data from *multiple datasets*. As we show in our experiments, our OSR-based AL method can automatically ignore samples that do not belong to the target dataset.

### 3.2. Active Learning System

Algorithm 1 summarizes our AL approach, which has two main components: a variational neural network (VNN) (Mundt et al., 2019b) that serves as our classifier and an OSR selection mechanism based on the loss function of the VNN. We discuss each component below.

**Algorithm 1:**
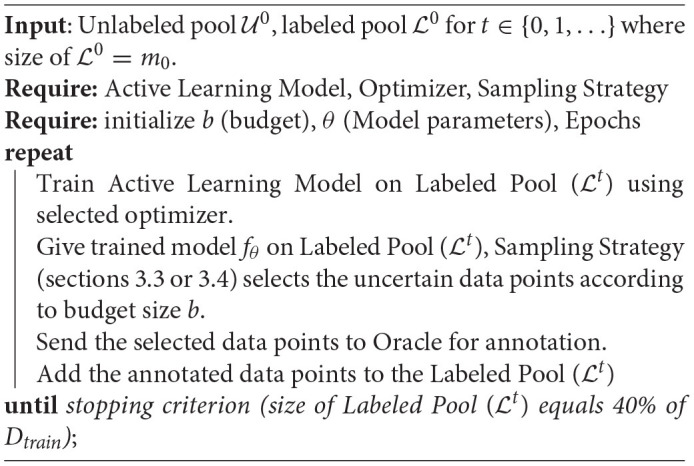
Active Learning

#### 3.2.1. Variational Neural Networks (VNNs)

Variational neural networks (VNNs) (Mundt et al., 2019b) are a supervised variant of β-variational autoencoders (β-VAE) (Higgins et al., 2017). The latter is itself a variant of VAEs (Doersch, 2016) but with a regularized cost function. That is, the cost function for a β-VAE consists of two terms: the reconstruction error, as with a regular VAE, and an *entanglement* penalty on the latent vector. This penalty forces the dimensions of the latent space to be as uncorrelated as possible, making them easier to interpret. A VNN combines the encoder-decoder architecture of a β-VAE with a probabilistic linear classifier (see Figure 1 for a visual representation). As such, its loss function includes a classification error, i.e., a supervised signal, in addition to the reconstruction and entanglement terms:

(1) $$\begin{array}{l} \begin{array}{l} L\left(\theta ,\phi ,\xi \right)={𝔼}_{q_{\theta }\left(z|x\right)}\left[\log p_{\phi }\left(x|z\right)+\log p_{\xi }\left(y|z\right)\right]\\ \;-\beta \text{KL}\left(q_{\theta }\left(z|x\right)||p\left(z\right)\right) \end{array} \end{array}$$

As detailed in Mundt et al. (2019b), θ, ϕ, and ξ are the parameters of the encoder, decoder, and classifier, resp., while *p*_ϕ_(***x***|***z***) and *p*_*ξ*_(***y***|***z***) are the reconstruction and classification terms. The last term is the entanglement penalty, which is given by the Kullback-Leibler divergence between the latent vector distribution and an isotropic Gaussian distribution.

As in Mundt et al. (2019b), we evaluated both the full framework discussed above (dubbed *M*_2_ in our experiments), which uses the loss function in Equation (1), and a simplified version (*M*_1_) without the reconstruction error:

(2) $$\begin{array}{l} L\left(\theta ,\xi \right)={𝔼}_{q_{\theta }\left(z|x\right)}\left[\log p_{\xi }\left(y|z\right)\right]-\beta \text{KL}\left(q_{\theta }\left(z|x\right)||p\left(z\right)\right) \end{array}$$

As our experiments show, both versions outperform the state of the art, but *M*_2_ achieves better results overall.

VNNs are especially suitable for OSR due to their *information bottleneck*. These networks are based on a variational approximation (Alemi et al., 2016) of the information bottleneck defined in Tishby et al. (2000). As detailed in Alemi et al. (2016), this variational approximation encourages a latent vector *z* to be predictive of the target label *y*, while at the same time encouraging *z* to “forget” the input *X*. Essentially it forces *z* to act like a minimal sufficient statistic of *X* for predicting *y*. In addition, in a VNN each input image gets mapped to a distribution rather than a unique latent vector, so it is unlikely that a small, idiosyncratic perturbations will pass through the information bottleneck. It is only when the input is very different from other inputs of same class that the latent vector will change significantly; this behavior leads to better generalization and robustness to noise. In Mundt et al. (2019b), it is shown using open-set experiments that VNNs produce more reliable confidence estimates compared to regular neural networks. This property plays a key role in our method for determining which samples to select from the unlabeled pool.

#### 3.2.2. Sample Selection

We wish to leverage the class disentanglement penalty defined in Equation (1). Specifically, our aim is to select *b* data points from the unlabeled pool U that the VNN is highly uncertain about. Following (Mundt et al., 2019a), in our experiments we investigated two sampling algorithms for OSR: *uncertainty sampling* and *Weibull distribution sampling*. The former is simpler, but the latter allows one to better reject outliers. We briefly describe each sampling strategy below.

### 3.3. Uncertainty Sampling

Here, we select a data point ***x***_*i*_ based directly on how uncertain the VNN is about it. Specifically, we rank all unlabeled samples by the value of the most likely class label and select the *b* samples with the lowest maximum values. Since the sum of class likelihoods is normalized, the value of the maximum class probability will approach one for highly certain samples and approach $\frac{1}{\left|C\right|}$, where |*C*| is the number of classes, for highly uncertain samples. In other words, the class likelihoods of uncertain samples have higher entropy than those for which the VNN is certain about.

### 3.4. Wiebull Distribution Sampling

As our experiments show, uncertainty sampling is suitable for active learning problems in which all unlabeled samples belong to known classes. However, for the case where the unlabeled pool also contains samples from unknown classes, we need a more robust way to exclude outliers. For this latter case, we employed the sampling procedure defined in Mundt et al. (2019a), which leverages a Wiebull distribution to estimate the model's uncertainty w.r.t a specific sample.

For completeness, here we will briefly outline the methodology proposed in Mundt et al. (2019a). Intuitively, it can be shown that it is useful to quantify the probability that a given data sample is an outlier, herein defined as a sample which is not sufficiently similar to those which have already been correctly classified. (Mundt et al., 2019a) show that this can be accomplished as follows. First, for each class, we compute the mean of the latent vectors of all samples that have been correctly predicted by the model. Second, we compute the distances from each class mean for all latent vectors, which (Mundt et al., 2019a) showed can be modeled with a Wiebull distribution. As such, a sample's likelihood under this distribution constitutes the minimum probability that the sample does ***not*** belong to any previously known class. In other words, the lower this value, the more likely that the sample is an outlier.

## 4. Experimental Results

We performed experiments on four image classification datasets—MNIST (LeCun et al., 2015), CIFAR-10 and CIFAR-100 (Krizhevsky, 2009), and FashionMNIST (Xiao et al., 2017)—following the methodology defined in section 3. Below, we first present our implementation details, then discuss our results.

### 4.1. Implementation Details

#### 4.1.1. Hardware

We carried out our experiments on a Dell Precision 7920R server with two Intel Xeon Silver 4110 CPUs, two GeForce GTX 1080 Ti graphics cards, and 128 GBs of RAM.

#### 4.1.2. Dataset Sizes and Budgets

**Figure 2:**
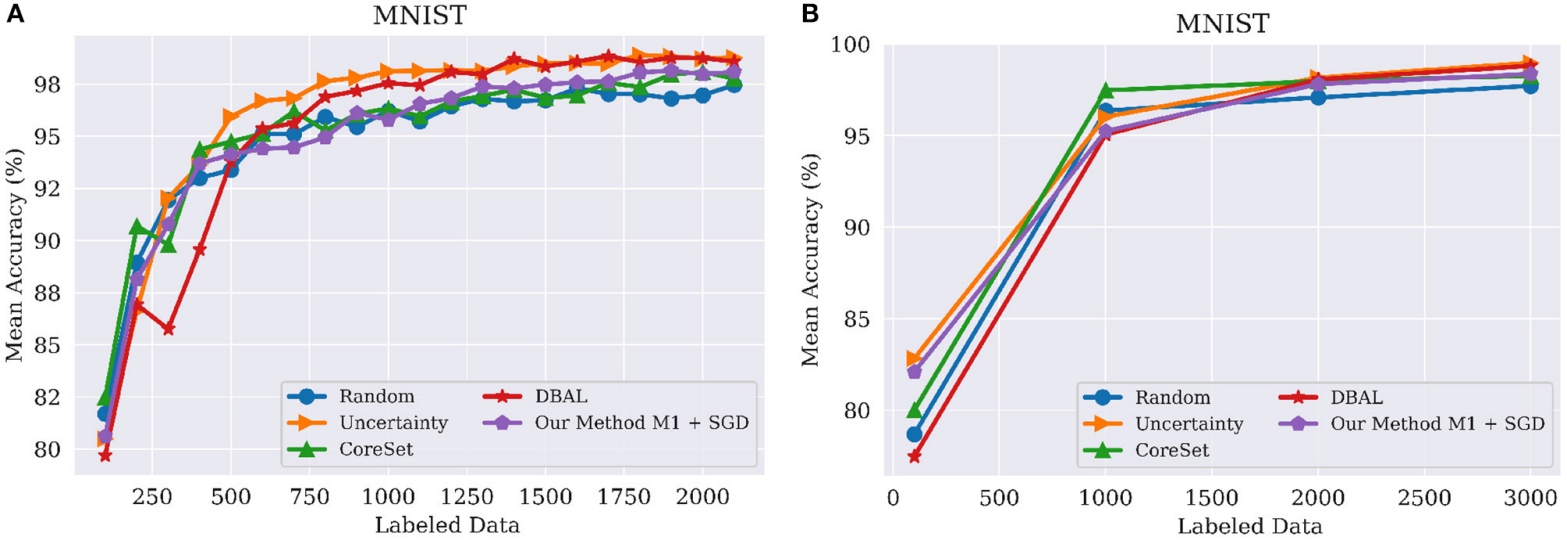
Performance on MNIST classification tasks using different query sizes for model *M*_1_. **(A)** Query batch size of 100; **(B)** Query batch size of 1,000 compared to Core-set (Sener and Savarese, 2017), DBAL (Gal et al., 2017), Random Sampling, and Uncertainty Sampling. M1 indicates our model with Encoder and Classifier. Best visible in color. Prior results adapted from Sinha et al. (2019).

As noted above, *budget* refers to the number of samples labeled by the oracle in each round of active learning. MNIST consists of 10,000 images for testing and 50,000 images for training out of which we used 100 for the initial labeled pool, 5,000 images as a validation set, and the remaining 44,900 images as part of the unlabeled pool. We used budgets of 100 and 1,000 samples for experiments (Figures 2A,B), resp. We used a similar setup for FashionMNIST. CIFAR-10 and CIFAR-100 also consist of 10,000 images for testing and 50,000 images for training. For these two datasets, we used 5,000 images as a validation set and the remaining 45,000 images were part of unlabeled and labeled pools. For CIFAR-10 and CIFAR-100, we used a budget of 2,500 images per round of active learning, up to 40% of the training data.

#### 4.1.3. Runs

For all datasets, we measured performance by computing the average accuracy across 5 independent runs.

#### 4.1.4. State of the Art Comparison

We compared our method against several recent AL approaches including Variational Adversarial Active Learning (VAAL) (Sinha et al., 2019), Core-Set (Sener and Savarese, 2017), Monte-Carlo Dropout (Gal and Ghahramani, 2016), Ensembles using Variation Ratios (Ensembles w. VarR) (Freeman, 1965; Beluch et al., 2018), Deep Bayesian AL (DBAL) (Gal et al., 2017), BatchBALD (Kirsch et al., 2019), and WAAL (Shui et al., 2020). As a baseline, we also included uniform random sampling (Random) since it remains a competitive strategy in the field of active learning.

#### 4.1.5. Architectures

For experiments on CIFAR-10 and CIFAR-100 we used a VGG16 network (Simonyan and Zisserman, 2014) as the encoder for both models, *M*_1_ and *M*_2_, and a decoder based on 14-layer residual networks (Zagoruyko and Komodakis, 2016; Higgins et al., 2017). We used latent vectors of size 60. As noted in section 3, the classifier consists of a single linear layer. For MNIST, we used a LeNET network (Lecun et al., 1998) as our encoder and a latent vector of size 60. Finally, for FashionMNIST we used the same convolutional network used by BatchBALD in their experiments (Kirsch et al., 2019).

#### 4.1.6. Optimization

We optimized all models using a mini-batch size of 128, a learning rate of 0.001, and a weight decay of 10^−5^. We tested two different optimizer, SGD and ADAM (Kingma and Ba, 2014), for both *M*_1_ and *M*_2_, for a total of four combinations:

- $M_{1}^{sgd}$ - Model *M*_1_ as shown in Equation (2) with SGD optimizer.
- $M_{1}^{adam}$ - Model *M*_1_ as shown in Equation (2) with Adam optimizer.
- $M_{2}^{sgd}$ - Model *M*_2_ as shown in Equation (1), with SGD optimizer.
- $M_{2}^{adam}$ - Model *M*_2_ as shown in Equation (1) with Adam optimizer.

#### 4.1.7. Oracle Queries

We defined a learning stage (i.e., a period of training between queries to the oracle) as lasting 150 epochs on CIFAR-10 and CIFAR-100 and 10 epochs on MNIST and FashionMNIST. At the completion of a stage, we requested labels for *b* images from the unlabeled pool. These were added to the labeled pool and used in the subsequent learning stages.

### 4.2. Image Classification Results

#### 4.2.1. MNIST

Our results were comparable with the state of the art on MNIST. However, as Figures 2A,B show, random sampling is already a highly successful strategy on MNIST, leaving little room for improvement on this dataset. In particular, as illustrated in Figure 2B, all methods obtained statistically similar results as the batch size increased. However, as shown in Figure 2A methods such as DBAL or Coreset have lower accuracies at the initial stages when using smaller batch sizes.

#### 4.2.2. FashionMNIST

**Figure 3:**
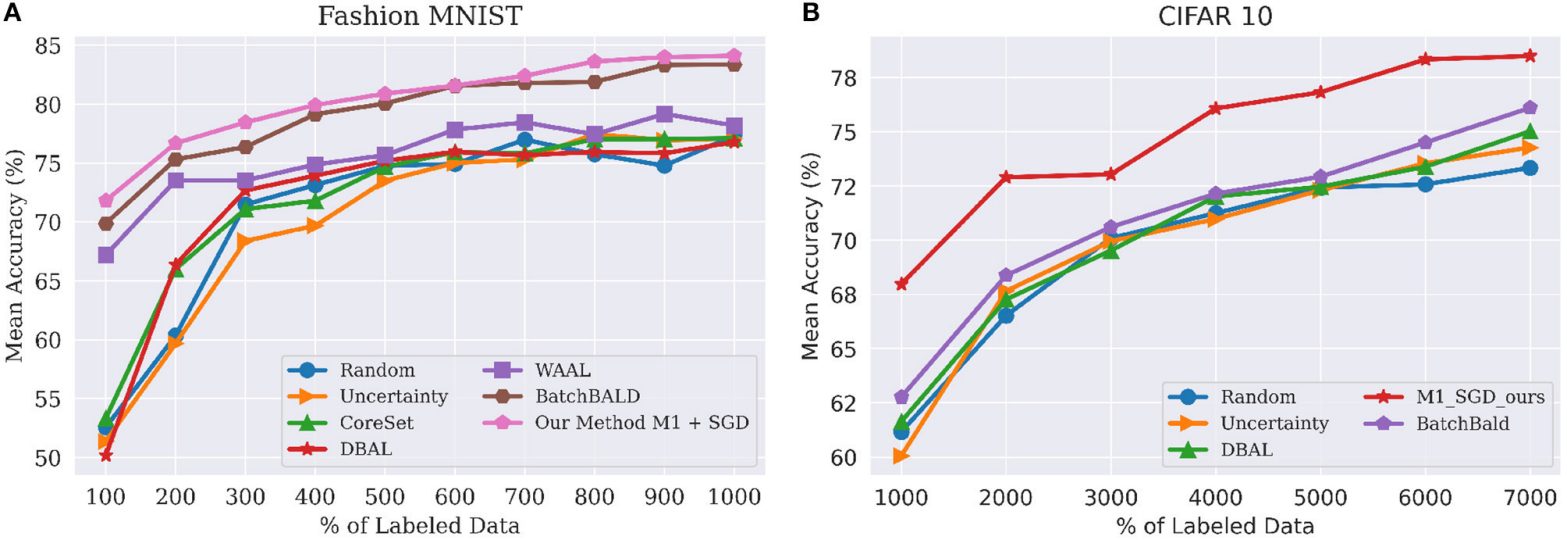
(**A**, left) Performance on FashionMNIST classification tasks using budget(*b*) of 100 for model *M*_1_ to WAAL (Shui et al., 2020), Core-set (Sener and Savarese, 2017), DBAL (Gal et al., 2017), BatchBALD (Kirsch et al., 2019), Random Sampling and Uncertainty Sampling. M1 indicates our model with Encoder and Classifier. (**B**, left) Performance on CIFAR-10 classification tasks using budget(*b*) of 1,000 for model *M*_1_. Best viewed in color.

For this dataset, we compared our approach against the existing state of the art, including BatchBALD, WAAL, CoreSet, and DBAL. As shown Figure 3, our method outperformed existing methods in each and every iteration of the active learning process. BatchBALD was the only method to achieve results similar to ours on this dataset; however, the inference time of our method is in the range of seconds, while for BatchBALD this inference time can range between minutes to hours depending on the budget size.

#### 4.2.3. CIFAR-10 and CIFAR-100

**Figure 4:**
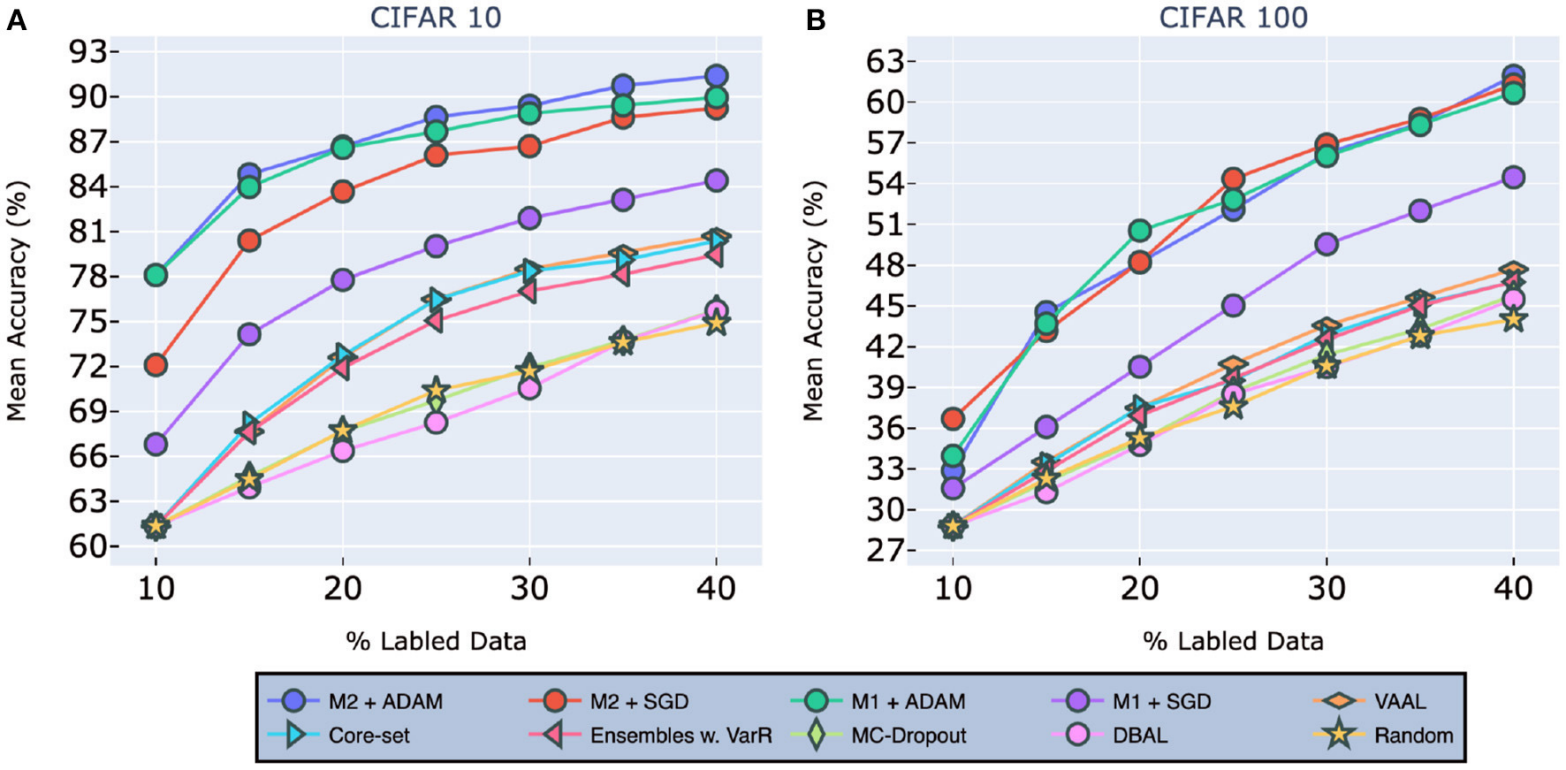
Performance on classification tasks for CIFAR-10 **(A)** and CIFAR-100 **(B)** compared to VAAL (Sinha et al., 2019), Core-set (Sener and Savarese, 2017), Ensembles w. VarR (Beluch et al., 2018), MC-Dropout (Gal and Ghahramani, 2016), DBAL (Gal et al., 2017), and Random Sampling. M1 indicates our model (2) and M2 indicates our model (1). All the legend names are in descending order of final accuracies. Best visible in color. Prior results adapted from Sinha et al. (2019).

As Figure 4 clearly shows, we achieved state-of-the-art performance by a considerable margin on both CIFAR-10 (left) and CIFAR-100 (right). On CIFAR-10, models [$M_{1}^{sgd},M_{1}^{adam},M_{2}^{sgd},M_{2}^{adam}$] achieved mean accuracies of [84.4, 89.24, 89.97, 91.4%], respectively. To put this in perspective, the original accuracy for this VNN using the entire CIFAR-10 dataset was 92.63%. VAAL came in second, with an accuracy of only 80.71%, followed by Core-Set with an accuracy of 80.37%, and then Ensemble w VarR at 79.465%. Random sampling, DBAL and MC-Dropout all trailed significantly behind other methods. Finally, we found that our models trained with ADAM, on average, outperform those trained with SGD.

In order to compare against BatchBALD, which has very high memory requirements, we had to use a slightly different experimental setup. Here, the size of labeled pool ($L_{0}$) was 5,000 and the budget(*b*) was 1,000. Under this setting, we compared our method against BatchBALD (Kirsch et al., 2019), DBAL (Gal et al., 2017), CoreSet (Sener and Savarese, 2017), Uncertainity sampling, and Random Sampling. These results are shown in Figure 3B. As before, our method outperformed all the existing method by a significant margin, similar to results obtained in Figure 4A.

For CIFAR-100, as shown in Figure 4, our models [$M_{1}^{sgd},M_{1}^{adam},M_{2}^{sgd},M_{2}^{adam}$] achieved mean accuracies of [54.47, 60.68, 61.25, 61.93%], resp. The original accuracy with the entire CIFAR-100 dataset was 63.14%. VAAL once again came in second, with an accuracy of 54.47 %, followed by Core-Set, and Ensemble w VarR. Here, it is worth nothing that most of existing methods fall in the same range of accuracies after training on 40% of the data.

### 4.3. Additional Experiments

In addition to our classification experiments, we replicated and extended the experiments of the same name put forth in Sinha et al. (2019) in order to investigate the robustness of our approach. Unless otherwise stated, we used CIFAR-100 for these experiments. Finally, we also tested our methods' ability to learn when the unlabeled pool contained out-of-distribution samples, a case which, to the best of our knowledge, cannot be handled by any existing methods.

#### 4.3.1. Effect of Biased Initial Pool

**Figure 5:**
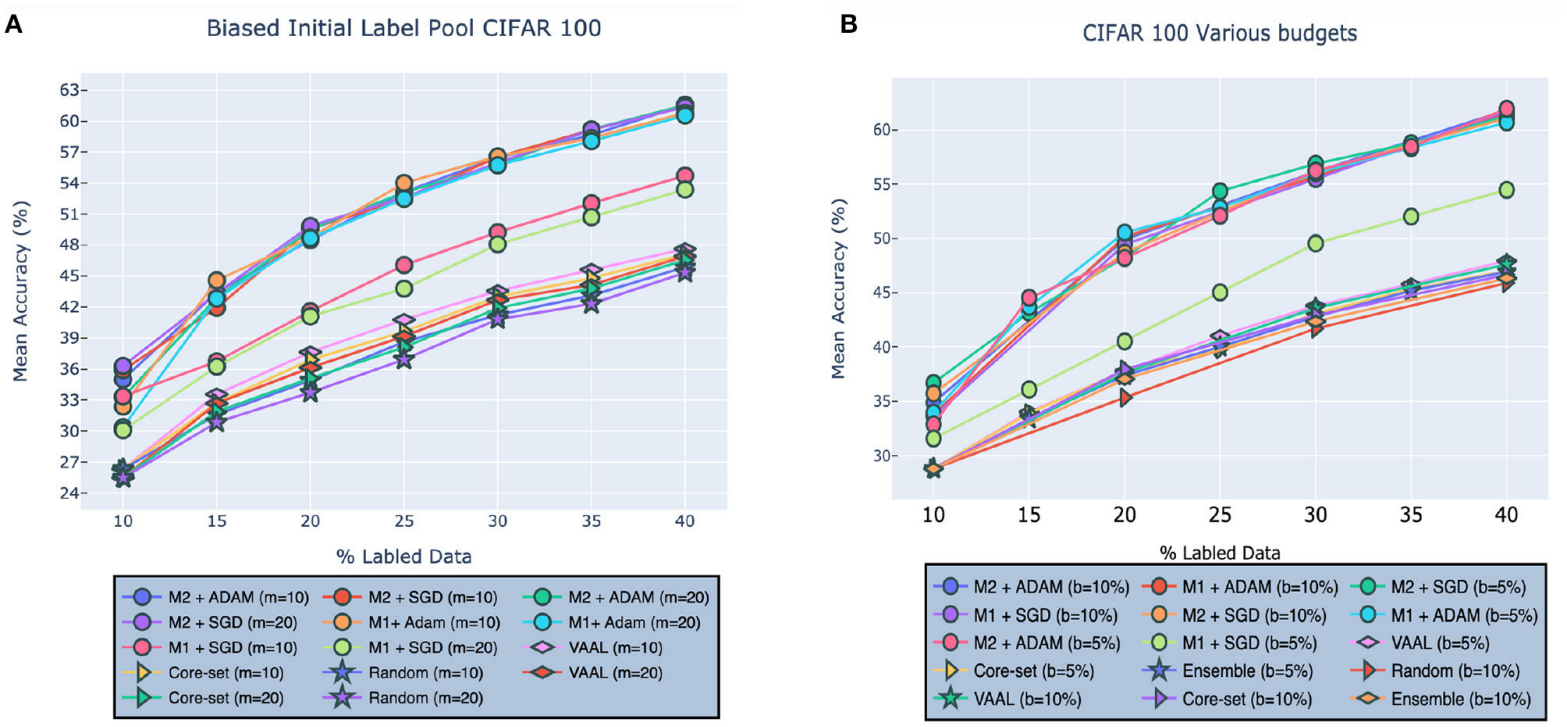
Robustness of our approach on CIFAR-100 given **(A)** biased initial labeled pool or **(B)** different budget sizes compared to VAAL (Sinha et al., 2019), Core-set (Sener and Savarese, 2017), Ensembles w. VarR (Beluch et al., 2018), MC-Dropout (Gal and Ghahramani, 2016), DBAL (Gal et al., 2017), and Random Sampling. M1 indicates our model (2) and M2 indicates our model (1). Best visible in color. Prior results adapted from Sinha et al. (2019).

We first investigated the effect of bias that may be present in the initial labeled pool, $L_{0}$. As stated in Sinha et al. (2019), bias can negatively impact the training of an active learner because it means that the initial labeled pool may not be representative of the true underlying data distribution. Unless explicitly accounted for, this will cause a system to learn an incomplete, or biased, model of the latent space. Following the protocol defined in Sinha et al. (2019), we removed all data points for *c* classes from $L_{0}$, thereby unbalancing the dataset and thus introducing bias. As shown in Figure 5A, our method outperformed VAAL, Core-set, and random sampling w.r.t selecting useful data points from classes that were underrepresented in the initial labeled pool. Models [$M_{1}^{sgd},M_{1}^{adam},M_{2}^{sgd},M_{2}^{adam}$] achieved accuracies of [53.35, 60.54, 61.36, 61.55%], respectively, when *c* = 20 and [54.72, 60.79, 61.53, 61.57%] when *c* = 10 (as noted above, *c* is the number of classes from which to exclude data). VAAL, by comparison, came in second, followed by Core-set, exhibiting accuracies [46.91, 46.55%] for *c*=20 and [47.10, 47.63%] for *c*=20, respectively. Random sampling achieved an accuracy of 45.33% for *c* = 10 and 45.87% for *c* = 20.

#### 4.3.2. Effect of Budget Size on Performance

In this section, we tested the effect of different budget sizes *b* on performance. Specifically, we investigated the effect of budgets of size *b* = 5% and *b* = 10%, referring to percentage of samples taken from $D_{train}$ at each stage of learning. As shown in Figure 5B, our model outperformed VAAL, Core-Set, Ensemble, and random sampling over both the budget sizes. VAAL comes in second followed by Core-set and Ensemble. Models [$M_{1}^{sgd},M_{1}^{adam},M_{2}^{sgd},M_{2}^{adam}$] achieve accuracies of [61.52, 61.57, 61.07, 61.82%] for *b* = 10 and [54.32, 60.68, 61.29, 61.9%] for *b* = 20.

#### 4.3.3. Noisy Oracle

**Figure 6:**
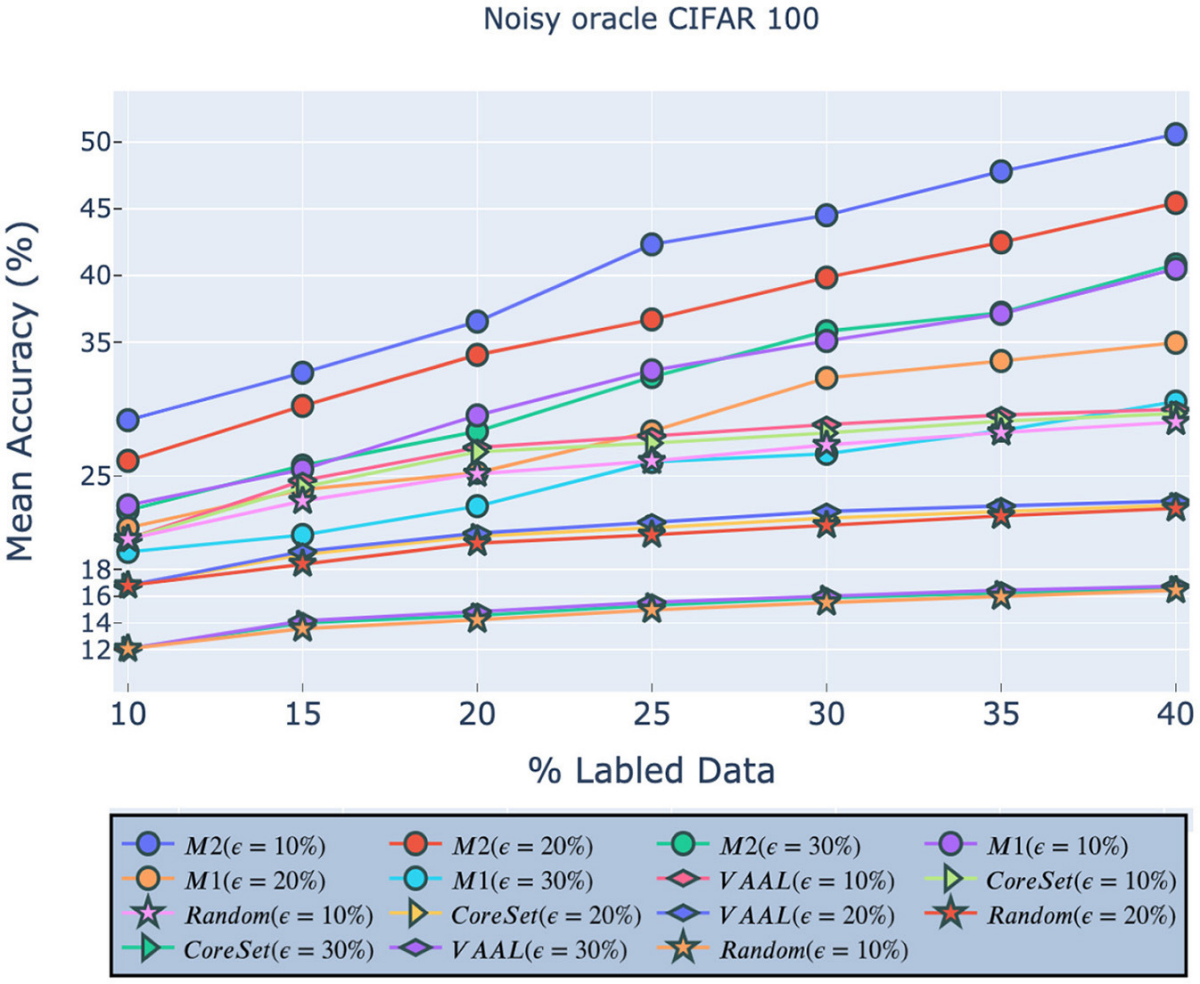
Robustness of our approach on CIFAR-100 given a noisy oracle. *M*_1_ indicates our model (2) and *M*_2_ indicates our model (1). All legend names are in descending order of final accuracies.

Next, we investigated the performance of our approach in the presence of noisy data caused by an inaccurate, or noisy oracle. As in Sinha et al. (2019), we assumed that incorrect labels can be caused by the natural ambiguity which exists between examples drawn from 2 separate classes, rather than adversarial attacks. CIFAR-100 has both classes and super-classes, so, following (Sinha et al., 2019), we randomly modified the labels of either 10, 20, or 30% of the samples by replacing them with a label from another class within the same super-class. As shown in Figure 6, our models consistently outperformed existing approaches *across all noise levels*. In other words, our *M*_1_ model with 30% noise was *more accurate* than VAAL, etc. with 10% noise.

#### 4.3.4. Sampling Time Analysis

We also replicated the sampling time analysis put forth in Sinha et al. (2019). Specifically, we measured the clock time of the “Sample Selection” box shown in Figure 1 for the various selection methods discussed in our results. We used the hardware listed in section 4.1 for all experiments. Table 1 shows that our method is competitive with other state-of-the-art techniques w.r.t. execution time, thereby offering strong empirical evidence that our method offers large performance advantages with minimal additional computation.

**Table 1:** Sampling time analysis: mean time to select a sample from the unlabeled pool of CIFAR-100.

**Method**	**Time (s)**
VAAL	10.69
**Uncertainty sampling**	**10.89**
DBAL	11.05
**Weibull sampling**	**20.41**
Ensembles w. VarR	20.48
Core-set	75.33
MC-dropout	83.65

*The bold values refer to our methods. The other values are for existing approaches*.

#### 4.3.5. Out-of-Distribution Samples in Unlabeled Pool

Finally, we also tested an extreme case of active learning in which data samples from other datasets are mixed into the current unlabeled pool. We used CIFAR-10 for these experiments. Here, we intentionally added 20% data (10,000 images) from other datasets to the unlabeled pool; thus, the network must distinguish not only between informative and non-informative samples but also distinguish *in-distribution* data samples from *out-of-distribution* samples. Whenever our model selected an OOD sample, the oracle discarded the sample, thus reducing the overall budget size. The discarded samples were placed back in the unlabeled pool (so the total number of OOD samples remained at 10,000).

**Figure 7:**
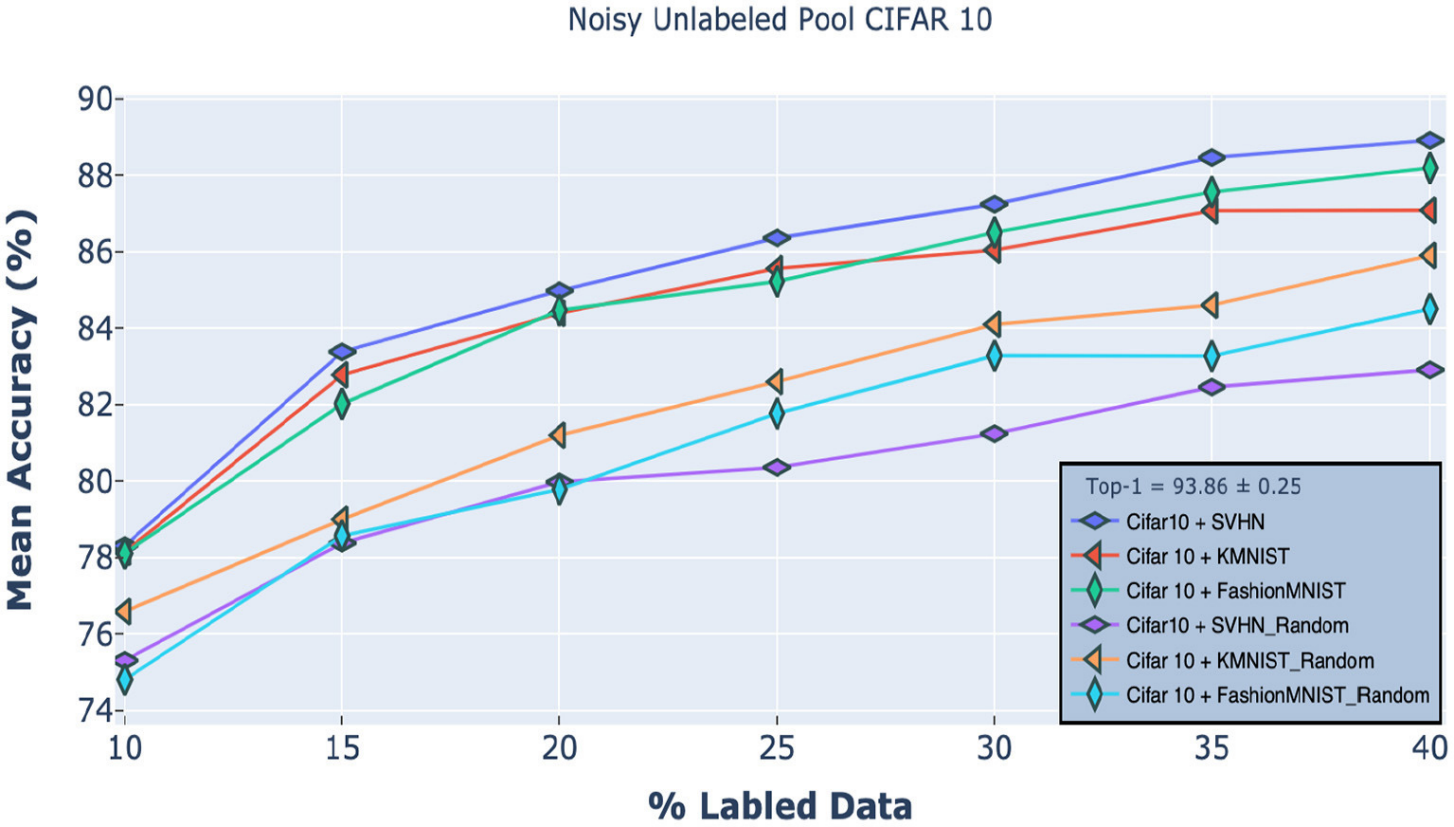
Robustness of our approach on CIFAR10 classification tasks when the unlabeled pool includes samples from either the SVHN, KMNIST, or FashionMNIST datasets. The first three curves used the *M*_2_ classifier, while the ones with the “Random” subscript used random sampling. Our results confirm that our approach significantly outperforms this baseline.

Figure 7 shows our *M*_2_ method's performance on CIFAR-10 when the unlabeled pool contained images from either SVHN, KMNIST, or FashionMNIST. Here, we used Weibull sampling (section 3.4) due to its better outlier rejection compared to uncertainty sampling. Specifically, we grouped the Weibull probabilities of the samples in the unlabeled pool into three categories: (1) *high-confidence* samples, which resemble the labeled pool; (2) *middle-confidence* samples, which are mostly samples from the target dataset that do not resemble the labeled pool; and (3) low-confidence samples, which are mostly samples from other datasets (OOD). Here, we want to sample middle confidence samples and ignore the rest. We empirically determined that samples with Weibull probabilities in the range of 0.4 to 0.8 corresponded to this middle confidence range and thus selecting only samples in this range yielded the best results. For comparison, we also tested random sampling as a baseline. Impressively, despite the presence of 20% OOD samples, our method significantly outperformed existing state-of-the-art methods trained on the regular unlabeled pool (Figure 4). And its performance, regardless of the second dataset, was only slightly below the standard *M*_2_ method.

### 4.4. Query Image Analysis

**Figure 8:**
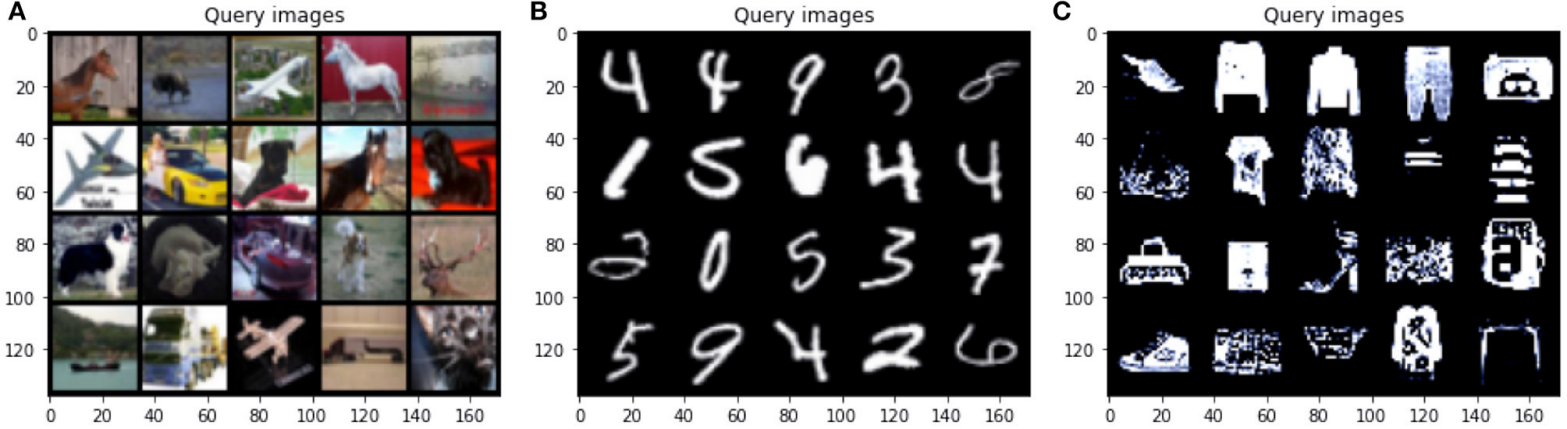
(**A**, left) Top 25 query images for CIFAR-10 selected in the first round of active learning using a budget(*b*) of 1,000 and model *M*_2_. (**B**, middle) Query images for MNIST and (**C**, right) FashionMNIST using a budget(*b*) of 100 and model *M*_1_.

Our experiments show that our uncertainty-based approach is highly successful at selecting informative samples across multiple datasets. To better understand what types of samples were deemed most informative under this scheme, in Figure 8, we plotted the top 25 samples selected from the unlabeled pool by our approach after the first round of active learning. This figure shows results for MNIST, CIFAR-10, and FashionMNIST. Overall, while there is some repetition in the chosen images (e.g., the top samples for MNIST included five fours), the full spectrum of samples is quite varied for all the datasets, suggesting that using uncertainty as a measure of informativeness may yield batch diversity as a side effect. We plan to investigate this hypothesis further in future work.

## 5. Conclusions and Future work

We have presented a novel approach for deep active learning using open-set recognition. To the best of our knowledge, we are the first to merge AL with OSR. Extensive experiments conducted over several image classification datasets have verified the effectiveness of our approach and established new state-of-the-arts benchmarks. Specifically, we empirically demonstrated that the samples most worth labeling are those which are most different from the current labeled pool. Training on such samples allows the model to learn features underrepresented in the existing training data. We extensively tested the robustness of our approach using different budget sizes, a noisy oracle, and an unlabeled pool comprised of multiple datasets. In future work, we plan to test our approach on continual learning problems, in which the system must learn to solve different problems over time. We also plan to test our method on other problems, including image segmentation and document classification.

## Data Availability Statement

The datasets presented in this study can be found in online repositories. The names of the repository/repositories and accession number(s) can be found below: https://github.com/jmandivarapu1/Deep-Active-Learning-via-Open-Set-Recognition.

## Author Contributions

JM and RE conceived of the presented idea. JM and BC carried out the experiments. JM, BC, and RE wrote the manuscript. All authors contributed to the article and approved the submitted version.

## Funding

This research was funded in part by NSF Award 1849946 and a grant from GoodAI Research.

## Conflict of Interest

The authors declare that the research was conducted in the absence of any commercial or financial relationships that could be construed as a potential conflict of interest.

## Publisher's Note

All claims expressed in this article are solely those of the authors and do not necessarily represent those of their affiliated organizations, or those of the publisher, the editors and the reviewers. Any product that may be evaluated in this article, or claim that may be made by its manufacturer, is not guaranteed or endorsed by the publisher.

## Footnotes

^1^As noted in Sinha et al. (2019), active learning can also refer to approaches that generate or synthesize novel samples. In this paper, however, we will only be concerned with pool-based active learning.
